# Evaluation of bone marrow lesion volume as a knee osteoarthritis biomarker - longitudinal relationships with pain and structural changes: data from the Osteoarthritis Initiative

**DOI:** 10.1186/ar4292

**Published:** 2013-09-10

**Authors:** Jeffrey B Driban, Lori Lyn Price, Grace H Lo, Jincheng Pang, David J Hunter, Eric Miller, Robert J Ward, Charles B Eaton, John A Lynch, Timothy E McAlindon

**Affiliations:** 1 Division of Rheumatology, Tufts Medical Center, 800 Washington Street, Box No 406, Boston, MA 02111, USA; 2 The Institute for Clinical Research and Health Policy Studies, Tufts Medical Center, and Tufts Clinical and Translational Science Institute, Tufts University, 800 Washington Street, Box No 63, Boston, MA 02111, USA; 3 Medical Care Line and Research Care Line; Houston Health Services Research and Development (HSR&D) Center of Excellence Michael E. DeBakey VAMC, 2002 Holcombe Boulevard, Houston, TX 77030, USA; 4 Section of Immunology, Allergy, and Rheumatology, Baylor College of Medicine, One Baylor Plaza, Houston, TX 77030, USA; 5 Department of Electrical and Computer Engineering, Tufts University, 101A Halligan Hall, Medford, MA 02155, USA; 6 Royal North Shore Hospital, Rheumatology Department and University of Sydney, Reserve Road, St Leonards, NSW 2065, Australia; 7 Department of Radiology, Tufts Medical Center, 800 Washington Street, Box No 299, Boston, MA 02111, USA; 8 Center for Primary Care and Prevention, Alpert Medical School of Brown University, 111 Brewster St, Pawtucket, RI 02860, USA; 9 Department of Epidemiology and Biostatistics, University of California at San Francisco, 185 Berry Street, San Francisco, CA 94107, USA

**Keywords:** Magnetic resonance imaging, symptoms, radiographs, joint space narrowing

## Abstract

**Introduction:**

Bone marrow lesion (BML) size may be an important imaging biomarker for osteoarthritis-related clinical trials and reducing BML size may be an important therapeutic goal. However, data on the interrelationships between BML size, pain, and structural progression are inconsistent and rarely examined in the same cohort. Therefore, we evaluated the cross-sectional and longitudinal associations of BML volume with knee pain and joint space narrowing (JSN).

**Methods:**

A BML volume assessment was performed on magnetic resonance images of the knee collected at the 24- and 48-month Osteoarthritis Initiative visits from a convenience sample of 404 participants in the progression cohort. During the same visits, knee pain was assessed with WOMAC pain scores and knee radiographs were acquired and scored for JSN. BML volume was summed to generate a total knee volume and an index tibiofemoral compartment volume (compartment with greater baseline JSN). Primary analyses included multiple linear regressions (outcome = pain, predictor = total knee BML volume) and logistic regressions (outcome = JSN, predictor = index tibiofemoral compartment BML volume).

**Results:**

This sample was 49% female with a mean age of 63 (9.2 standard deviation (SD)) years, and 71% had radiographic osteoarthritis in the study knee. Larger baseline BMLs were associated with greater baseline knee pain (*P *= 0.01), the presence of JSN at baseline (odds ratio (OR) = 1.50, 95% confidence interval (CI) = 1.23 to 1.83), and JSN progression (OR = 1.27, 95%CI = 1.11 to 1.46). Changes in total knee BML volume had a positive association with changes in knee pain severity (*P *= 0.004) and this association may be driven by knees that were progressing from no or small baseline BMLs to larger BMLs. In contrast, we found no linear positive relationship between BML volume change and JSN progression. Instead, regression of medial tibiofemoral BML volume was associated with JSN progression compared to knees with no or minimal changes in BML volume (OR = 3.36, 95%CI = 1.55 to 7.28). However, follow-up analyses indicated that the association between JSN progression and BML volume change may primarily be influenced by baseline BML volume.

**Conclusion:**

Large baseline BMLs are associated with greater baseline knee pain, the presence of JSN at baseline, and disease progression. Additionally, BML regression is associated with decreased knee pain but not a reduced risk of concurrent JSN progression.

## Introduction

**Figure 1:**
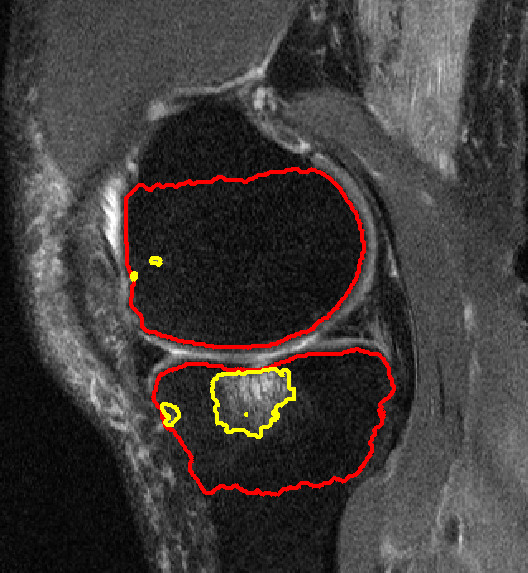
**Bone marrow lesion (BML) in the medial tibia**. The red lines identify the bone boundary and the yellow lines surround areas of high signal intensity. The three small regions (two in femur, one in tibia) would be excluded from analyses since they do not appear on more than one image.

Knee osteoarthritis (OA) is commonly characterized by periarticular bone changes [1-6]. For example, bone marrow lesions (BMLs; see Figure 1) are related to OA severity and may predict OA progression [1-8]. Furthermore, incident or enlarging BMLs are associated with incident and increased knee pain [9-11] especially among knees without OA or when the baseline BML size is small and the increase in size is large [9-11]. In addition to changes in knee pain, several studies using semi-quantitative outcomes or two-dimensional measurements suggest that an increase in BML size is associated with cartilage loss; however, the temporal order of these pathologic changes remains unclear [5,12-14].

Based on these associations, BML size may be an important imaging biomarker for clinical trials [15] and reducing BML size may be an important therapeutic goal for disease modification [16,17]. For BMLs to function in this role, changes in their size should reflect changes in disease activity, however, data on the inter-relationships between BML size, pain, and structural progression are inconsistent [9,10,12,14] and rarely examined in the same cohort. It may be beneficial to explore the longitudinal association between BML size and structural progression as well as knee pain in the same cohort.

Therefore, the purposes of these analyses were to evaluate the cross-sectional and longitudinal associations between BML volume and knee pain as well as joint space narrowing (JSN), with an emphasis on exploring decreases in BML size. We hypothesized that larger baseline BML sizes will be associated with greater baseline knee pain and the presence of JSN as well as greater increases in knee pain and JSN progression over 24 months. Furthermore, we hypothesized that changes in BML size would have a positive linear association with changes in knee pain and JSN progression. Therefore, we anticipated that decreases in BML size would be associated with decreases in knee pain and reduced odds of JSN progression. The results of these analyses may provide further evidence to support or refute the utility of BML reduction as a therapeutic goal and BML volume as an imaging biomarker for clinical trials.

## Methods

To assess the associations between BML volume and knee pain as well as JSN we used images and data obtained from the Osteoarthritis Initiative (OAI). The OAI is a multicenter observational cohort study of knee OA that collected longitudinal clinical and image data [18] as well as biospecimens from 4,796 participants over an eight-year follow-up period. The primary variables (knee pain, JSN, and BML volume) were from the 24-month and 48-month OAI visits. OAI data are available for public access [19].

### Participant selection

The OAI participants were classified at baseline into three subcohorts, which included the progression (*n *= 1,389) subcohort that was characterized by participants with symptomatic radiographic knee OA in at least one knee. Symptomatic radiographic knee OA was defined as a knee with a definite osteophyte (Osteoarthritis Research Society International (OARSI) Atlas [20] osteophyte grade 1 to 3) and symptoms ('pain, aching or stiffness on most days of the month in the last year').

The Bone Ancillary Study recruited participants (*n *= 629) in the OAI progression subcohort during their 30- or 36-month OAI visits. An inclusion criterion for this ancillary study was a willingness to undergo additional knee imaging. Exclusion criteria were contraindication for magnetic resonance (MR) imaging and the presence of bilateral knee replacements. For these analyses, we focused on participants in the Bone Ancillary Study with MR imaging at the 24- and 48-month OAI visits (*n *= 478). We then excluded knees that had imaging artifact (for example, motion) or the bone segmentation program failed to delineate the bone borders (*n *= 442). From this sample, we selected the first 404 knees as a convenience sample. MR images from these visits were selected because of the time period overlaps with the data collected for the Bone Ancillary Study. This study received ethical approval from each OAI clinical site (Memorial Hospital of Rhode Island Institutional Review Board, Ohio State University's Biomedical Sciences Institutional Review Board, University of Pittsburgh Institutional Review Board, and University of Maryland Baltimore - Institutional Review Board), the OAI coordinating center (Committee on Human Research at University of California, San Francisco), as well as the Institutional Review Board at Tufts Medical Center and Tufts University Health Sciences Campus. All participants provided informed consent to the OAI and the Bone Ancillary Study.

### Magnetic resonance imaging

All BML measurements were performed using sagittal intermediate-weighted, turbo spin echo, fat-suppressed MR sequences (field of view = 160 mm, slice thickness = 3 mm, skip = 0 mm, flip angle = 180 degrees, echo time = 30 ms, recovery time = 3200 ms, 313 × 448 matrix (interpolated to 512 × 512), phase encode superior/inferior, × resolution = 0.357 mm, and y resolution = 0.511 mm) [18]. The measured images were acquired at the 24- and 48-month OAI visits with one of four identical Siemens (Erlangen, Germany) Trio 3-Tesla MR systems and a USA Instruments (Aurora, OH, USA) quadrature transmit-receive knee coil at the four OAI clinical sites.

For these analyses, we focused on the primary OAI knee, which was the right knee unless there was a contraindication for MR imaging (for example, the presence of metal), in which case, the left knee was the primary OAI knee. This knee was selected because, according to the OAI protocol, it underwent a complete set of OAI MR sequences, while the contralateral knee had an abbreviated MR scan to reduce participant burden. Therefore, the primary OAI knee was not always the knee with symptomatic OA.

### Semi-automated BML segmentation

Two readers measured BML volume with a semi-automated segmentation method that detects, extracts, and quantifies the structure of BMLs based on the sagittal intermediate weighted, turbo spin echo, fat-suppressed MR sequence. We have previously reported the construct validity of this method with OAI images by demonstrating that increases in BML volume were associated with cartilage loss and BML volumes differed across the Boston Leeds Osteoarthritis Knee Score [21]. This semi-automated segmentation method has been described in more detail elsewhere [21]; but briefly, the only manual step required a reader to use a custom graphical user interface (MATLAB, MathWorks, Inc., Natick, MA, USA) to manually identify the crude boundaries of the tibia and femur in each slice of the MR imaging data set by marking multiple points along the articular surface. For the border furthest from the articular surface, the reader marked the bone just prior to the epiphyseal line or at the edge of bone and soft tissue. In addition, we omitted the central slices from the analyses (that is, the middle nine slices; 2.7 cm) to focus on BMLs adjacent to the chondral surface and to improve reliability. The program automatically refined the initial estimate to more precisely identify the bone boundaries (see red lines in Figure 1). Next, the program automatically performed a thresholding and curve evolution process twice to segment the areas of high signal intensity, which may represent a probable BML (see yellow lines in Figure 1). We then used two criteria to eliminate the false-positive regions and to operationally define a BML: (1) the distance between a BML to the articular surface should be no more than 10 mm [6,22,23] and (2) a BML should span more than one MR image. BML volumes were calculated for four discrete regions: medial femur, lateral femur, medial tibia, and lateral tibia.

Intra-reader and inter-reader reliability were assessed for the two readers and the details of those methods have been previously described [21]. Intra-reader reliability for BML change was good to excellent for reader one (intraclass correlation coefficient (ICC) (3,1 model) = 0.79 to >0.99, *n *= 10) and reader two (ICC (3,1 model) = 0.95 to 0.96, *n *= 12). Inter-reader reliability for BML volume change was good for the lateral femur and tibia as well as the medial femur (ICC (2,1 model) = 0.83 to 0.93) but low for the medial tibia BML volume change (ICC (2,1 model) = 0.59). To ensure consistency between readers a third investigator reviewed all of the BML segmentations. The third investigator was responsible for ensuring that the bone segmentation was consistent across time and knees. The same reader always measured the baseline and follow-up images to avoid inter-reader error in the BML change measurements.

### Knee pain

Knee pain was assessed in the primary OAI knee with a knee-specific WOMAC (Western Ontario and McMaster Universities) pain score. The WOMAC pain score was derived from five 5-point Likert-based questions that inquire about knee pain over the last seven days when performing different activities (walking, stairs, in bed, seated or lying down, and standing). The WOMAC pain score is a well-validated pain assessment that is commonly used for pain related to knee OA [24]. WOMAC pain scores at the 24- and 48-month OAI visits are publicly available (Files: AllClinical03_SAS (version 3.4) and AllClinical06_SAS (version 6.2)) [19].

### Knee radiographs

Weight-bearing, bilateral, fixed-flexion, posterior-anterior knee radiographs were obtained at the 24- and 48-month OAI visits. Central readers, who were blinded to sequence, scored the paired images for medial and lateral JSN grade (0 to 3) using the OARSI Atlas [20] as well as within OARSI grade narrowing [25]. The agreement for these readings (read-reread) was good (weighted kappa (intra-rater reliability) = 0.75 to 0.88). These JSN scores are publicly available (Files: kXR_SQ_BU03_SAS(version 3.4) and kXR_SQ_BU06_SAS (version 6.20) [19].

### Confirmatory analyses

To verify the primary results for structural (JSN) progression we used data from a clinical trial of vitamin D among patients with knee OA (*n *= 103) [6,26]. The advantage of this data set was that we had manually measured BMLs, which allowed us to verify our findings using a different BML measurement method and a different structural outcome measure - cartilage thickness [6]. The methods to measure BMLs and cartilage thickness have been reported previously [6,26]. Briefly, femoral and tibial cartilage was manually segmented by one reader in the index tibiofemoral compartment (compartment with greater JSN; ICC >0.99). Furthermore, one reader measured the longest cross-sectional diameter of each BML in three planes to approximate BML volume (ICC = 0.90). Approximate BML volumes were then summed to form a compartment-specific BML volume.

### Statistical analyses

Demographic and anthropometric descriptive statistics were calculated for the samples used in each analysis (data is publicly available [19]). We used chi-square tests and independent-sample *t *tests to determine if these participant characteristics were different between our OAI study sample and the remainder of the progression subcohort that was not included in our primary analyses. Furthermore, we calculated the descriptive characteristics of baseline BML volume and BML volume change (follow-up volume minus baseline volume).

Among the full cohort, we explored the distribution of total BML volume change (total BML volume = medial femur + lateral femur + medial tibia + lateral tibia) when stratified by tertiles based on baseline total BML volume (no or small BML volume, moderate BML volume, large BML volume). Total BML volume change was stratified into quartiles but the middle two quartiles were collapsed. Therefore, the lowest quartile represented knees with BML regression, the middle two quartiles represented knees with no or minimal changes in BML volumes, and the highest quartile represented knees with BML progression (enlargement).

#### Total bone marrow lesion volumes and knee pain

Three multiple linear regression models were used to evaluate the association between WOMAC pain (continuous variable), as an outcome, and total BML volume (continuous variable) while controlling for sex, weight (at 24-month visit), height (at 24-month visit), and age (at 24-month visit). The first model was used to assess the association between baseline WOMAC pain (continuous variable) and baseline total BML volume (continuous variable). The second and third models explored the associations between WOMAC pain change (continuous variable) and baseline total BML volume and total BML volume change (continuous variables), respectively. Prior to the analyses we verified a linear relationship between WOMAC pain (baseline and change) and total BML volume (baseline and change).

To further explore the associations between WOMAC pain change and total BML volume change we assessed the associations stratified among tertiles based on baseline BML volume. Based on diagnostic tests (for example, DFFITS, Cook's D) that yielded poor diagnostics for the linear regression models within tertiles, we opted to perform robust regression models.

#### Unicompartmental bone marrow lesion volumes and joint space narrowing

Three logistic regression models were used to evaluate the association between JSN, as an outcome, and BML volume in the index tibiofemoral compartment. The first model was used to assess the association between the presence of JSN at baseline (dichotomous variable) and baseline BML volume (continuous variable). The second and third models explored the associations between JSN progression (dichotomous variable) and baseline BML volume (continuous variable) and BML volume change (three classifications), respectively. The JSN analyses were limited to the index tibiofemoral compartment, which was defined as the compartment with greater JSN at baseline. JSN progression was defined as any increase in OARSI JSN scores, including within grade changes [25]. For models with JSN progression as the outcome we excluded knees with severe JSN (OARSI JSN score = 3) since these knees could not progress. The BML volume was the sum of the femur and tibia BML volumes in the index compartment. In the logistic regressions, we controlled for sex, body mass index (at 24-month visit), and age (at 24-month visit). We adjusted for body mass index instead of weight and height because of the limited number of knees with JSN progression.

We evaluated the point estimates across quartiles of each continuous independent variable (that is, age, body mass index, baseline BML volume, and BML volume change) to verify if they had a linear relationship with the outcomes. Age did not have a linear relationship with the outcomes; therefore, we converted age to a binary variable (<65 years, ≥65 years). Body mass index also did not have a linear relationship with the outcomes and was converted to a binary variable (<30 kg/m^2^, ≥30 kg/m^2^). Finally, BML volume change did not have a linear relationship with the presence of JSN progression. Therefore, we analyzed the association between JSN progression and BML volume change quartiles with the middle two quartiles collapsed. Therefore, the lowest quartile represented knees with BML regression, the middle two quartiles represented knees with no or minimal changes in BML volumes (reference group), and the highest quartile represented knees with BML progression (enlargement). Sensitivity analyses were performed among knees with primarily medial tibiofemoral JSN.

Based on the results of the analyses noted above, we used classification and regression trees (CART) [27,28] to determine whether baseline BML volume or BML volume change was more influential in predicting JSN progression over 24 months. JSN progression was used as the outcome in CART analysis and the predictors were baseline BML volume, BML volume change, age, sex, and body mass index. One advantage of CART is that for a given set of predictors, CART searches for the optimal cut point that best discriminates between participants with and without JSN progression. Statistical significance was defined as *P *≤0.05. All analyses were performed in SAS 9.3 (SAS Institute Inc., Cary, NC, USA) with the exception of the CART analyses, which were conducted in R 2.15.0 using the rpart function.

#### Confirmatory follow-up analyses

To verify the primary structural findings, we performed a logistic regression model to examine the association between manually measured BML size change (classified as BML regression (change <-3.2 cm^3^), no change (change ± 3.2 cm^3^), or BML progression (change >3.2 cm^3^) [6]) and the outcome of tibial cartilage thickness derived from manual cartilage segmentation (stratified into tertiles). The model for femoral cartilage thickness was omitted from the results because it did not meet the assumptions of linearity (score test for the proportional odds assumption; *P *= 0.02).

## Results

**Figure 2:**
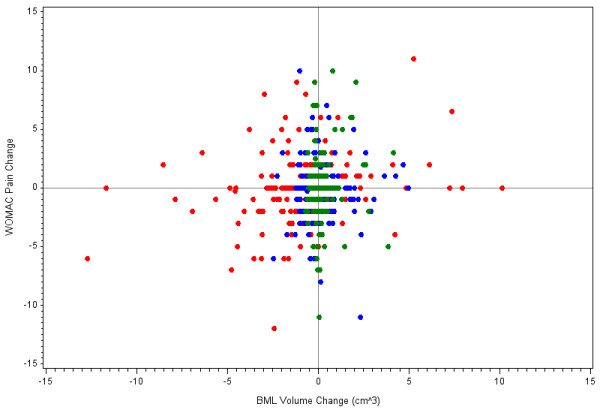
**Scatter plot of WOMAC pain change by bone marrow lesion (BML) volume change stratified by tertiles (colors)**. Tertiles, based on baseline BML volume, had average baseline total knee BML volumes of 0.6 ± 0.2 cm^3^, 1.6 ± 0.5 cm^3^, and 5.5 ± 2.9 cm^3^, respectively.

The descriptive characteristics of the cohort (*n *= 404) and the remainder of the progression subcohort that was not included in these analyses, are reported in Table 1. The average baseline total knee BML volume ranged from 0.1 to 15.5 cm^3 ^and the total knee BML volume change ranged from -12.7 to 10.2 cm^3 ^(see Figure 2). The distribution of BML volume change stratified by baseline BML volume is described in Table 2. Most knees with no to moderate baseline total BML volume showed minimal or no BML change. In contrast, the majority of knees with large baseline total BML volume experienced BML regression over 24 months.

**Table 1:** Descriptive characteristics of study sample (*n *= 404) and other Progression Subcohort members not analyzed (*n *= 986).

	Study sample mean (SD) or n (%)	Remainder of Progression subcohort^1^ mean (SD) or n (%)
Females	199 (49%)	594 (60%)*
Age (years)	62.9 (9.2)	63.7 (9.0)
Weight (kg)	85.0 (15.4)	85.9 (16.4)
Height (m)	1.7 (0.1)	1.7 (0.1)
Right knee	390 (97%)	n/a^2^
Kellgren and Lawrence grade ≥2	287 (71%)	539 (78%)*
Primarily lateral tibiofemoral OA	62 (15%)	110 (16%)
Baseline WOMAC pain score	3.3 (3.4)	4.1 (3.9)*
Baseline WOMAC pain score = 0	109 (27%)	189 (24%)
Change in WOMAC pain score	0.0 (3.0)	-0.3 (3.4)
Change in WOMAC pain score = 0	102 (25%)	174 (24%)
Total knee BML volume (baseline; cm^3^)	2.6 (2.7)	n/a
Total knee BML volume (change; cm^3^)	-0.2 (2.1)	n/a

Note: 1. Numbers for the remainder of the progression subcohort may vary due to missing data. 2. For the remainder of the progression subcohort we analyzed the right knee for knee-specific outcomes. *P <0.05 for chi-square or independent-sample *t *tests comparing study sample to the remainders of the progression subcohort. BML, bone marrow lesion; n/a, not applicable; OA, osteoarthritis; SD, standard deviation.

**Table 2:** Distribution of total BML volume change when stratified by tertiles based on total baseline BML volume

		Total BML volume change (by quartile with middle two quartiles collapsed)		
	
Total baseline BML volume	Total BML volume change Median (min, max) cm^3^	'BML regression' (Range: -12.7 to -0.8 cm^3^) n (% of baseline tertile)	'Minimal or no BML change' (Range: -0.8 to 0.4 cm^3^) n (% of baseline tertile)	'BML progression' (Range: 0.4 to 10.2 cm^3^) n (% of baseline tertile)
'No or Small BML volume' (Range: 0.1 to 1.0 cm^3^)	0.0 (-0.7, 4.2)	0 (0.0%)	108 (80.6%)	26 (19.4%)
'Moderate BML volume' (Range: 1.0 to 2.7 cm^3^)	-0.1 (-2.5, 5.0)	24 (17.8%)	68 (50.4%)	43 (31.9%)
'Large BML volume' (Range: 2.7 to 15.5 cm^3^)	-1.1 (-12.7, 10.2)	77 (57.0%)	26 (19.3%)	32 (23.7%)

The division of total baseline bone marrow lesion (BML) volume and total BML volume change was based on tertiles. *n *= 404.

### Total bone marrow lesion volumes and knee pain

The associations between WOMAC pain and total BML volume are described in Table 3 (*n *= 404). Larger total baseline BML volumes were associated with greater knee pain. Furthermore, BML volume change was positively associated with change in knee pain (for example, an increase in BML volume was associated with increased knee pain).

**Table 3:** Cross-sectional and longitudinal association between BML volume and WOMAC pain volume

	B (SE)*	(*P *value)
Outcome: WOMAC Pain (baseline)		
BML volume (baseline)	0.16 (0.06)	0.014
Outcome: WOMAC pain (change)		
BML volume (baseline)	-0.01 (0.06)	0.874
BML volume (change)	0.21 (0.07)	0.004

*All models adjusted for sex, weight, height, and age. *n *= 404. BML, bone marrow lesion; B, parameter estimate; SE, standard error.

Since prior research suggested the association between BML change and pain may be more pronounced among knees progressing from no or small BMLs at baseline, we further explored the association between WOMAC pain change and BML volume change by conducting stratified analyses by baseline BML volume (tertiles). The robust regression models indicated that total BML volume change and WOMAC pain change were significantly related (estimate = 0.65, standard error = 0.25, *P *= 0.009) only in the first tertile (no BMLs or BMLs <1.0 cm^3^).

### Unicompartmental bone marrow lesion volumes and joint space narrowing

Among 375 knees with BML and JSN data, 246 (66%) had JSN at baseline. This sample included 25 knees with severe JSN (grade 3) that were excluded from analyses of JSN progression. Among the remaining 350 knees, 68 (19%) knees had JSN progression over 24 months.

Larger baseline BML volume was associated with the presence of baseline JSN in the same tibiofemoral compartment (odds ratio (OR) = 1.50 (95% confidence interval (CI) = 1.23 to 1.82), c-statistic = 0.69). Knees without baseline JSN had a median baseline BML volume of 0.49 cm^3 ^(range 0.06 to 5.76 cm^3^). Knees with baseline JSN had a median baseline BML volume of 1.13 cm^3 ^(range 0.06 to 14.95 cm^3^).

Larger baseline BML volume was associated with JSN progression in the same tibiofemoral compartment (odds ratio = 1.28 (95% CI = 1.12 to 1.47), c-statistic = 0.70). Knees without JSN progression had a median baseline BML volume of 0.61 cm^3 ^(range 0.06 to 14.57 cm^3^). Knees with JSN progression had a median baseline BML volume of 1.32 cm^3 ^(range 0.08 to 14.95 cm^3^).

Table 4 shows the association between BML volume change and JSN progression. Overall, BML volume change was not significantly associated with JSN progression (*P *= 0.11). However, there was a trend that BML regression and BML progression may be associated with increased odds of JSN progression relative to knees with no or minimal changes in BML volume.

**Table 4:** Association between joint space narrowing (JSN) progression and bone marrow lesion (BML) volume change

		JSN progression			
				
Rank for change in BML volume	Median (min, max) cm^3^	No	Yes	Odds ratio* (95% CI)	Overall *P *value
Medial or lateral JSN progression (*n *= 350)					
BML regression (*n *= 87)	-0.97 (-12.59, -0.46)	66 (76%)	21 (24%)	1.86 (0.96 to 3.60)	
No to minimal BML change (*n *= 176) (middle two quartiles collapsed)	-0.05 (-0.46, 0.24)	150 (85%)	26 (15%)	Reference	0.11
BML progression (*n *= 87)	0.73 (0.25, 10.02)	66 (76%)	21 (24%)	1.79 (0.92 to 3.46)	
Medial JSN progression (*n *= 301)					
Medial TF BML regression (*n *= 75)	-0.97 (-9.48, -0.46)	53 (74%)	19 (26%)	3.07 (1.44 to 6.57)	
No to minimal medial TF BML change (*n *= 151) (middle two quartiles collapsed)	-0.08 (-0.46, 0.23)	130 (89%)	16 (11%)	Reference	0.01
Medial TF BML progression (*n *= 75)	0.65 (0.23, 4.76)	58 (81%)	14 (19%)	1.62 (0.73 to 3.61)	

All changes are based on change between the 24- and 48-month Osteoarthritis Initiative visits. *Odds ratios are adjusted for sex, baseline age (<65 years, ≥65 years), and baseline body mass index (<30 kg/m^2^, ≥30 kg/m^2^). 95% CI, 95% confidence interval; TF, tibiofemoral.

Sensitivity analyses were performed among knees with more severe medial tibiofemoral JSN than lateral tibiofemoral JSN. Among 317 knees, 188 (59%) had JSN at baseline. This sample included 16 knees with severe JSN (grade 3) that were excluded from analyses of JSN progression. Among the remaining 301 knees, 49 (16%) knees had JSN progression over 24 months. Overall, we found that medial tibiofemoral BML volume change was associated with medial JSN progression (*P *= 0.01). Specifically, BML regression in the medial tibiofemoral compartment was statistically associated with increased odds of medial tibiofemoral JSN progression (Table 4).

Based on the results above, we tried to determine whether baseline BML volume or BML volume change was more influential in modeling JSN progression over 24 months. In CART analyses the first split was based on baseline BML volume, suggesting that this is the variable best able to discriminate between those who did and did not have JSN progression (see Figure S1 in Additional file 1). The analysis classified participants with baseline BML volume less than 0.95 cm^3 ^as non-progressors (including 22 who had JSN progression). For participants with baseline BML volume greater than 0.95 cm^3^, classification into the progressor and non-progressor groups depended on further splits based on change in BML volume, baseline BML volume, and age, resulting in 17 participants correctly classified as progressors, 5 incorrectly classified as progressors, and 29 incorrectly classified as not progressing.

### Confirmatory analyses: approximate BML volume and cartilage change

In the confirmatory data set, 21 knees experienced BML regression in the index tibiofemoral compartment, 49 knees experienced no change (reference group), and 33 had BML progression. While BML change was not associated with tibial cartilage thickness (in tertiles, *P *= 0.08) there was a trend indicating BML regression (OR = 2.72, 95% CI = 1.03 to 7.12) and BML progression (OR = 2.06, 95% CI = 0.88 to 4.57) were associated with greater odds of cartilage thickness loss.

## Discussion

Bone marrow lesion volume may have some utility as an imaging biomarker for assessing knee OA severity and predicting knee OA progression. However, reduction in BML volume, which is associated with decreases in knee pain, does not appear to indicate improvement in other aspects of OA. Therefore, the use of BML volume as a surrogate endpoint is not supported by this study.

Despite a lack of support for BML volume as a surrogate endpoint, this study demonstrates why assessing BML volume at baseline may be beneficial. Based on these analyses, we hypothesize that baseline BML size may be more important than BML change when examining the association between BMLs and JSN progression. These longitudinal findings may indicate that once a knee experiences a large BML the joint has been structurally compromised, at least for the next 24 months. BML regression may represent changes that lead to decreased knee pain (for example, reduced edema or fibrovascular tissue) but may not reflect recovery of the bone structural integrity (for example, periarticular bone mineral density, trabecular morphometry), which may take longer to remodel.

Consistent with what we found, cross-sectional [1,29-31] and longitudinal analyses [2,6,8,11] have highlighted that larger baseline BML size is associated with greater baseline knee pain and structural damage as well as disease progression [2,4-8,13,14]. Baseline BML size may be particularly important when assessing the associations between changes in BML size and disease progression. For example, our results provide additional evidence that the association between changes in knee pain and BML volume may be more pronounced among knees with no or small baseline BMLs [11]. Additionally, we found that in the association between JSN progression and BMLs it may be that baseline BML size is more important than BML volume change. These findings may be related to the underlying structural changes associated with BMLs. BMLs are characterized by altered subchondral bone structure (for example, decreased mineral content, fibrosis, edema, and altered trabecular morphometry) [32-38]. Perhaps progression from no or small baseline BML coincides with these changes leading to an increase in symptoms and JSN progression. Therefore, large BMLs are associated with abnormal bone structures. Once the bone has been compromised with a large BML, a reduction in BML size may reflect changes in the bone that can adapt quickly and may be related to knee symptoms (for example, fibrosis, edema) but not reflect changes in structural integrity (for example, mineral content, altered trabecular morphometry), which may relate to risk of JSN progression and take longer to recover.

This hypothesis is contingent on our findings that BML regression, which is typical of larger baseline BMLs, was associated with JSN progression. To verify these results, we analyzed a different data set using manual measurements of approximate BML volume and cartilage changes. The confirmatory analyses, while not significant, indicated similar trends as reported in the primary analyses. Furthermore, these results may be in agreement with prior work from the MOST Study which reported that 28.1 to 29.3% of regions with decreased BMLs had increases in cartilage defect scores compared to 27.2% of those with stable BMLs or 6.0% of those with no BMLs at baseline or follow-up (these latter two groups represent a similar definition as our reference group with no to minimal changes) [14]. Based on the results from the MOST Study [14] and the current analyses, BML regression may not be an optimal surrogate endpoint. To verify that BML regression may not be an optimal surrogate endpoint, we replicated the analyses with JSN progression using percent change in BML volume to determine if percent change performed differently in the statistical models than absolute change but these analyses were not statistically significant (data not shown). Based on the CART analysis, the analyses with percent BML change may have been null because smaller baseline BMLs (<0.95 cm^3^) would have considerable percent change even though the absolute change was minimal (-0.8 to 0.4 cm^3^). This further supports the hypothesis that BML regression (absolute change or percent change) may not be an optimal surrogate endpoint. Therefore, if we want to reduce the risk of cartilage loss or JSN then BML prevention may be a more appropriate goal than BML regression

While the study highlights the importance of baseline BML size, it raises questions about interpreting BML volume change that the current study could not address. First, the semi-automated approach may include measurement error associated with incorrectly identifying areas of high signal intensity in the bone as BMLs. To reduce the risk of incorrectly identifying areas of high signal intensity as BMLs we established rules *a priori *(that is, BMLs must be on consecutive images, BMLs must be within 10 mm of articular cartilage, the central nine slices were omitted). These rules systematically exclude some BMLs, particularly in the central region of the knee. For the pain analyses, the exclusion of centrally located BMLs would likely lead to underestimates of the strength of the association between BMLs and pain. However, for the structural analyses, it is unlikely that omitting the central slices would influence our results. Hernandez-Molina *et al. *[39] reported that central BMLs were not associated with cartilage loss unless they extended into subchondral region in the index compartment, which would have been detected with our segmentation method. Future research with semi-quantitative readings may help us better understand the implications of omitting some areas of high signal intensity and measurement error associated with this semi-automated approach. If the measurement error was random, then the error would have increased the likelihood of null results. In addition to measurement error, it is important to acknowledge that these results may be specific to the MR sequence we measured. Future research may be warranted to confirm these findings on other MR sequences.

Another limitation to these analyses is that there were only two time points measured. One study has suggested that BML change may occur within six to twelve weeks [15]. Therefore, it is difficult to determine when the BML volume change occurred within the two-year follow-up period. If the BML volume change primarily occurred in the last few months of the follow-up period, then there may not have been adequate time to observe the benefits of BML regression. These findings highlight the importance of future research to measure BML volume change over multiple time periods, with shorter intervals between assessments, to evaluate how fluctuations in BML size relate to disease progression. Studies with multiple time points may be key to understanding whether the joint remains compromised once a large BML emerges. This may be particularly relevant for clinical trials that aim to reduce BML size [40]; particularly since these studies may be ideal for determining the clinical meaningfulness of the association between change in BML volume and change in pain.

Besides being limited to two time points, this study was also limited by 68 cases of JSN progression among 375 knees. This limitation may have been greater if BML changes were classified based on semi-quantitative changes, which offers a limited range for scoring change. In this study, BML volume was advantageous because it provided a continuous outcome that could be stratified into groups (for example, tertiles, quartiles) and within these groups have sufficient heterogeneity for regression models. Notwithstanding this advantage, longer follow-up periods or larger sample sizes may help increase the number of knees with JSN progression in future studies.

Finally, we used a convenience sample selected from the OAI progression subcohort, which may introduce a selection bias. The OAI progression subcohort included individuals from four clinical sites (Rhode Island, Maryland, Pennsylvania, Ohio) with at least one knee with symptomatic knee OA. The OAI is not a population-based sample but is a well-described study sample with a rich dataset including MR imaging, clinical variables, and biospecimens. Furthermore, within this subcohort we analyzed 404 knees, which had a similar age, weight, and height to the rest of the progression cohort but included less females, less knees with radiographic OA, and knees with less WOMAC pain scores (Table 1). This may introduce some bias but, despite this limitation, our analyses with joint symptoms complement the existing literature and our structural analyses using the OAI data was consistent with the confirmatory analyses using the clinical trial data.

Despite these limitations, this study may demonstrate that once a knee experiences a large BML the joint has been structurally compromised, at least for 24 months. Additional research will be needed to test hypotheses for why this may be the case (for example, despite BML regression on MR images the bone structure remains compromised). Based on these hypotheses, prevention of large BMLs may be a more desirable therapeutic goal than trying to reduce BML size.

## Conclusions

In conclusion, this study provides further evidence that baseline BML size in an important imaging biomarker associated with greater baseline knee pain, the presence of structural damage at baseline, and OA progression. However, this study did not support the use of BML volume change as a surrogate endpoint. We found that BML regression was associated with decreases in knee pain but not a change in risk for structural progression over 24 months. Based on secondary analyses (CART analysis), small baseline BMLs may not be associated with JSN progression, regardless of BML change, but among knees with larger baseline BMLs the chance of JSN progression is influenced by baseline BML size and change in BML size.

## Abbreviations

BML: bone marrow lesion; CART: classification and regression trees; CI: confidence interval; ICC: intraclass correlation coefficient; JSN: joint space narrowing; MR: magnetic resonance; OA: osteoarthritis; OAI: Osteoarthritis Initiative; OARSI: Osteoarthritis Research Society International; OR: odds ratio.

## Competing interests

The authors declare that they have no competing interests.

The Role of Bone in Knee Osteoarthritis Progression is supported by NIH/NIAMS (grant 1R01AR054938). The OAI is a public-private partnership comprised of five contracts (N01-AR-2-2258; N01-AR-2-2259; N01-AR-2-2260; N01-AR-2-2261; N01-AR-2-2262) funded by the National Institutes of Health, a branch of the Department of Health and Human Services, and conducted by the OAI Study Investigators. Private funding partners include Pfizer, Inc.; Novartis Pharmaceuticals Corporation; Merck Research Laboratories; and GlaxoSmithKline. Private sector funding for the OAI is managed by the Foundation for the National Institutes of Health. This manuscript has received the approval of the OAI Publications Committee based on a review of its scientific content and data interpretation. This work was supported in part by the Houston VA HSR&D Center of Excellence (HFP90-020). The views expressed in this article are those of the authors and do not necessarily represent the views of the Department of Veterans Affairs.

## Authors' contributions

JBD contributed to the conception and design, acquisition of data, analysis and interpretation of data, drafting/revisions of the manuscript, as well as final approval of the manuscript. LLP contributed to the conception and design, analysis and interpretation of data, drafting/revisions of the manuscript, as well as final approval of the manuscript. GHL contributed to the conception and design, analysis and interpretation of data, drafting/revisions of the manuscript, as well as final approval of the manuscript JP contributed to the conception and design, acquisition of data, analysis and interpretation of data, drafting/revisions of the manuscript, as well as final approval of the manuscript. DJH contributed to the conception and design, acquisition of data, analysis and interpretation of data, drafting/revisions of the manuscript, as well as final approval of the manuscript. EM contributed to the conception and design, acquisition of data, analysis and interpretation of data, drafting/revisions of the manuscript, as well as final approval of the manuscript. RJW contributed to the conception and design, acquisition of data, analysis and interpretation of data, drafting/revisions of the manuscript, as well as final approval of the manuscript CBE contributed to the conception and design, analysis and interpretation of data, drafting/revisions of the manuscript, as well as final approval of the manuscript. JAL contributed to the conception and design, acquisition of data, analysis and interpretation of data, drafting/revisions of the manuscript, as well as final approval of the manuscript. TEM contributed to the conception and design, analysis and interpretation of data, drafting/revisions of the manuscript, as well as final approval of the manuscript. All authors read and approved the final manuscript.

## Supplementary Material

Additional file 1 **Figure S1**. Classification and regression trees intended to classify participants with and without joint space narrowing progression. The analysis classified participants with baseline bone marrow lesion (BML) volume less than 0.95 cm^3 ^as participants without joint space narrowing (JSN) progression. For participants with baseline BML volume greater than 0.95 cm^3^, classification into the progressor and non-progressor groups depended on further splits based on change in BML volume, baseline BML volume, and age. Click here for file
