# Supplementary material for: Evaluation of bone marrow lesion volume as a knee osteoarthritis biomarker - longitudinal relationships with pain and structural changes: data from the Osteoarthritis Initiative
Source: Arthritis Res Ther. 2013 Sep 10;15(5):R112. doi: 10.1186/ar4292 (PMC3978948; doi:10.1186/ar4292)
Supplement: Additional file 1 — Figure S1. Classification and regression trees intended to classify participants with and without joint space narrowing progression. The analysis classified participants with baseline bone marrow lesion (BML) volume less than 0.95 cm3 as participants without joint space narrowing (JSN) progression. For participants with baseline BML volume greater than 0.95 cm3, classification into the progressor and non-progressor groups depended on further splits based on change in BML volume, baseline BML volume, and age. [file ar4292-S1.PPT]

## Slide 1
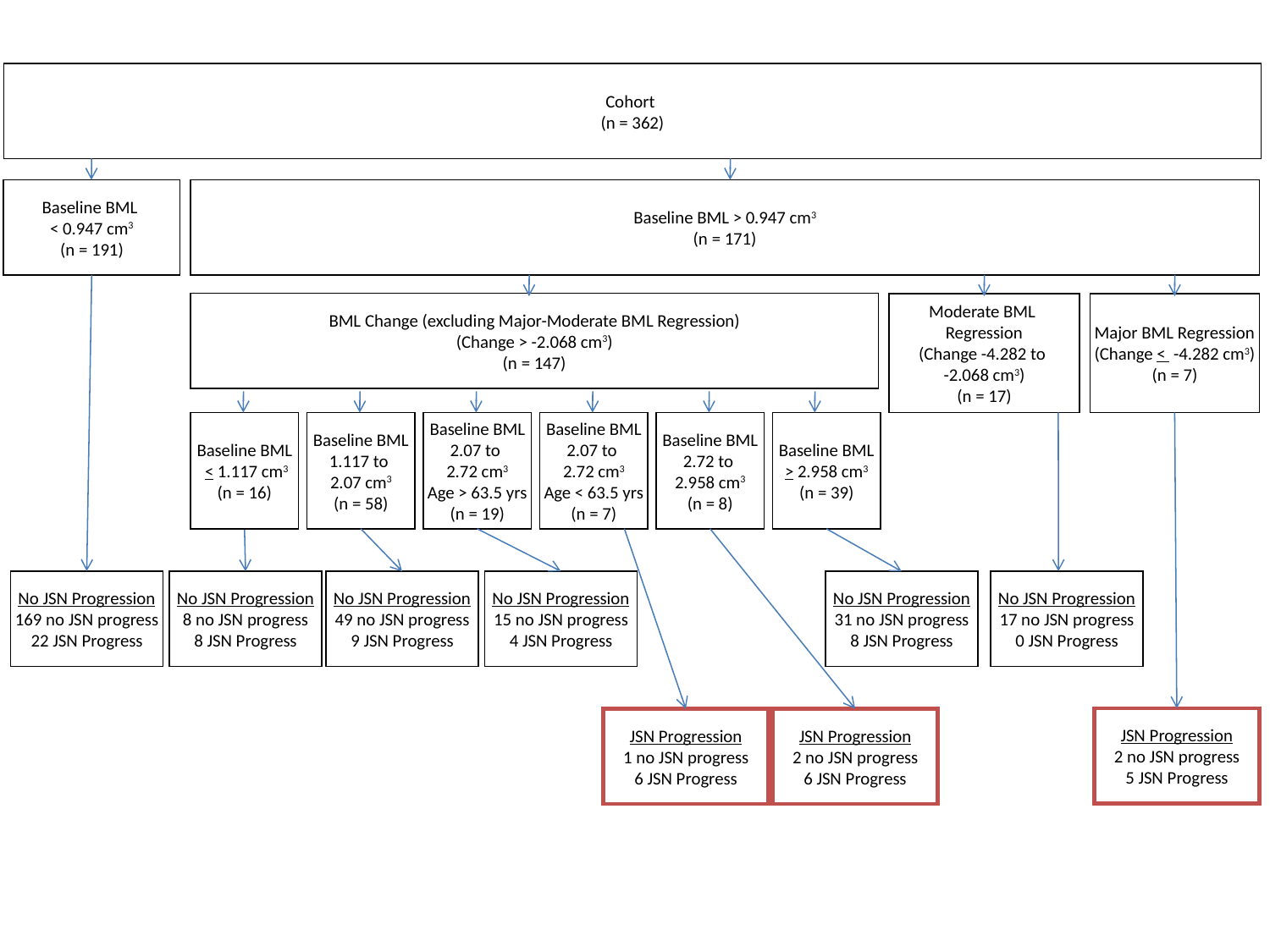

Cohort
(n = 362)
Baseline BML
< 0.947 cm3
(n = 191)
Baseline BML > 0.947 cm3
(n = 171)
BML Change (excluding Major-Moderate BML Regression)
(Change > -2.068 cm3)
(n = 147)
Moderate BML
Regression
(Change -4.282 to
-2.068 cm3)
(n = 17)
Major BML Regression
(Change < -4.282 cm3)
(n = 7)
Baseline BML
 < 1.117 cm3
(n = 16)
Baseline BML
1.117 to
2.07 cm3
(n = 58)
Baseline BML
2.07 to
2.72 cm3
Age > 63.5 yrs
(n = 19)
Baseline BML
2.07 to
2.72 cm3
Age < 63.5 yrs
(n = 7)
Baseline BML
2.72 to
2.958 cm3
(n = 8)
Baseline BML
> 2.958 cm3
(n = 39)
No JSN Progression
169 no JSN progress
22 JSN Progress
No JSN Progression
15 no JSN progress
4 JSN Progress
No JSN Progression
17 no JSN progress
0 JSN Progress
No JSN Progression
8 no JSN progress
8 JSN Progress
No JSN Progression
49 no JSN progress
9 JSN Progress
No JSN Progression
31 no JSN progress
8 JSN Progress
JSN Progression
2 no JSN progress
5 JSN Progress
JSN Progression
1 no JSN progress
6 JSN Progress
JSN Progression
2 no JSN progress
6 JSN Progress
